# (*E*)-*N*′-(2-Hydr­oxy-4-methoxy­benzyl­idene)isonicotinohydrazide monohydrate

**DOI:** 10.1107/S1600536808029619

**Published:** 2008-09-24

**Authors:** San-Jun Peng, Hai-Yun Hou

**Affiliations:** a College of Chemistry and Biological Engineering, Changsha University of Science and Technology, Changsha 410076, People’s Republic of China; b College of Environmental and Chemical Engineering, Xi’an Polytechnic University, Xi’an 710048, People’s Republic of China

## Abstract

The title compound, C_14_H_13_N_3_O_3_·H_2_O, was prepared by the reaction of 4-methoxy­salicylaldehyde and isonicotinohydrazide in ethanol. The Schiff base mol­ecule is not planar and has an *E* configuration with respect to the methyl­idene unit. The dihedral angle between the benzene and pyridine rings is 36.8 (2)°. In the mol­ecule there is an intra­molecular O—H⋯N hydrogen bond involving the hydroxyl substituent and the N atom of the 2-hydr­oxy-4-methoxy­benzyl­idene unit. In the crystal, the mol­ecules are linked through inter­molecular O—H⋯O, O—H⋯N and N—H⋯O hydrogen bonds, forming layers parallel to the *bc* plane.

## Related literature

For bond-length data, see: Allen *et al.* (1987 ▶). For background on the biological properties of hydrazones, see: El-Tabl *et al.* (2008 ▶); Chen *et al.* (2008 ▶); Alvarez *et al.* (2008 ▶); Ventura & Martins (2008 ▶); Kalinowski *et al.* (2008 ▶). For related structures, see: Peng & Hou (2008 ▶); Shan *et al.* (2008 ▶); Fun *et al.* (2008 ▶); Yehye *et al.* (2008 ▶); Ejsmont *et al.* (2008 ▶); Han *et al.* (2006 ▶); Lu *et al.* (2008 ▶).
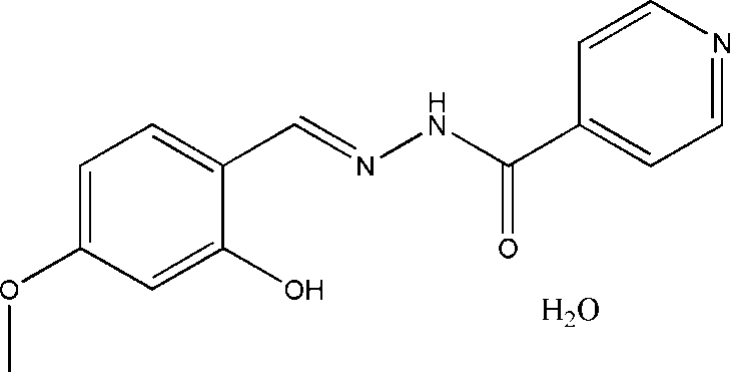


## Experimental

### 

#### Crystal data

C_14_H_13_N_3_O_3_·H_2_O
*M* *_r_* = 289.29
Monoclinic, 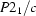

*a* = 7.299 (4) Å
*b* = 12.537 (6) Å
*c* = 14.808 (7) Å
β = 96.281 (8)°
*V* = 1346.9 (11) Å^3^
*Z* = 4
Mo *K*α radiation
μ = 0.11 mm^−1^
*T* = 298 (2) K
0.23 × 0.23 × 0.22 mm

#### Data collection

Bruker SMART 1000 CCD area-detector diffractometer
Absorption correction: multi-scan (*SADABS*; Bruker, 2001 ▶) *T*_min_ = 0.976, *T*_max_ = 0.977
7804 measured reflections
3041 independent reflections
2129 reflections with *I* > 2σ(*I*)
*R*_int_ = 0.030

#### Refinement

*R*[*F* ^2^ > 2σ(*F* ^2^)] = 0.045
*wR*(*F* ^2^) = 0.112
*S* = 1.03
3041 reflections
201 parameters
4 restraints
H atoms treated by a mixture of independent and constrained refinement
Δρ_max_ = 0.18 e Å^−3^
Δρ_min_ = −0.25 e Å^−3^

### 

Data collection: *SMART* (Bruker, 2007 ▶); cell refinement: *SAINT* (Bruker, 2007 ▶); data reduction: *SAINT*; program(s) used to solve structure: *SHELXTL* (Sheldrick, 2008 ▶); program(s) used to refine structure: *SHELXTL*; molecular graphics: *SHELXTL*; software used to prepare material for publication: *SHELXTL*.

## Supplementary Material

Crystal structure: contains datablocks global, I. DOI: 10.1107/S1600536808029619/su2062sup1.cif

Structure factors: contains datablocks I. DOI: 10.1107/S1600536808029619/su2062Isup2.hkl

Additional supplementary materials:  crystallographic information; 3D view; checkCIF report

## Figures and Tables

**Table 1:** Hydrogen-bond geometry (Å, °)

*D*—H⋯*A*	*D*—H	H⋯*A*	*D*⋯*A*	*D*—H⋯*A*
O1—H1⋯N1	0.82	1.92	2.644 (2)	146
O4—H4*B*⋯O2^i^	0.853 (9)	2.072 (10)	2.924 (2)	176 (2)
O4—H4*A*⋯N3^ii^	0.861 (9)	1.971 (10)	2.832 (2)	178 (2)
N2—H2⋯O4^iii^	0.903 (9)	2.024 (11)	2.915 (2)	169 (2)

Symmetry codes: (i) 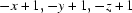; (ii) 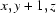; (iii) 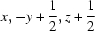.

**Fig. 1.**
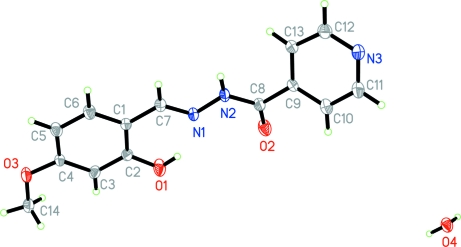
The molecular structure of compound (I), with 30% probability displacement ellipsoids for non-H atoms.

**Fig. 2.**
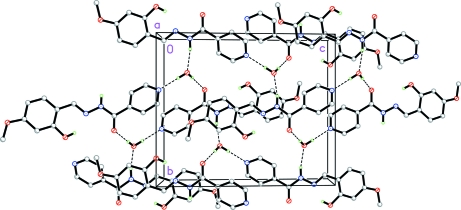
The crystal packing diagram of compound (I), viewed along the a axis. Hydrogen bonds are shown as dashed lines.

## supplementary crystallographic information

### Comment

Hydrazones derived from the reactions of aldehydes with hydrazides show potential biological properties (El-Tabl *et al.*, 2008; Chen *et al.*, 2008; Alvarez *et al.*, 2008; Ventura & Martins, 2008; Kalinowski *et al.*, 2008). In the last few years, a large number of hydrazones have been reported (Peng & Hou, 2008; Shan *et al.*, 2008; Fun *et al.*, 2008; Yehye *et al.*, 2008; Ejsmont *et al.*, 2008). As a continuous study, the crystal structure of the title compound, (I), is reported in this paper.

The molecular structure of compound (I) is illustrated in Fig. 1. It consists of a Schiff base molecule and a water molecule of crystallization. The C7═N1 bond length of 1.276 (2) Å indicates a typical C═N double bond. The Schiff base molecule has an E configuration with respect to the methylidene unit (C7═N1), as observed in similar compounds (Han *et al.*, 2006; Lu *et al.*, 2008). In the molecule there is an intramolecular O-H···N hydrogen bond involving the hydroxyl substituent and the N-atom of the 2-hydroxy-4-methoxybenzylidene moiety (Table 1). The dihedral angle between the benzene and pyridine rings is 36.8 (2)°, indicating the molecule is not planar. The bond lengths are in normal ranges (Allen *et al.*, 1987).

In the crystal structure, symmetry related molecules are linked through intermolecular O—H···O, O—H···N and N—H···O hydrogen bonds (Table 1), forming layers parallel to the *bc* plane (Fig. 2).

### Experimental

4-Methoxysalicylaldehyde (0.152 g, 1 mmol) was dissolved in 95% ethanol (50 ml), then isonicotinohydrazide (0.137 g, 1 mmol) was added slowly to the solution, and the mixture was heated at reflux with continuous stirring for 1 h. The solution was cooled to room temperature, yielding colorless crystallites. Recrystallization from a 95% ethanol yielded block-like single crytals of compound (I).

### Refinement

H-atoms H2, H4A and H4B were located in a difference Fourier map and refined isotropically, with N—H, O—H and H···H distances restrained to 0.90 (1), 0.85 (1) and 1.37 (2) Å, respectively, and with U_iso_(H) set at 0.08 Å^2^. The other H atoms were placed in calculated positions and treated as riding atoms with C—H = 0.93 - 0.96 Å, O—H = 0.82 Å, and i>U_iso_(H) = 1.2U_eq_(C) and 1.5U_eq_(O1 and C14).

### Crystal data

**Table:**

C_14_H_13_N_3_O_3_·H_2_O	*F*(000) = 608
*M**_r_* = 289.29	*D*_x_ = 1.427 Mg m^−^^3^
Monoclinic, *P*2_1_/*c*	Mo *K*α radiation, λ = 0.71073 Å
Hall symbol: -P 2ybc	Cell parameters from 1237 reflections
*a* = 7.299 (4) Å	θ = 2.4–24.5°
*b* = 12.537 (6) Å	µ = 0.11 mm^−^^1^
*c* = 14.808 (7) Å	*T* = 298 K
β = 96.281 (8)°	Block, colorless
*V* = 1346.9 (11) Å^3^	0.23 × 0.23 × 0.22 mm
*Z* = 4	

### Data collection

**Table:**

Bruker SMART 1000 CCD area-detector diffractometer	3041 independent reflections
Radiation source: fine-focus sealed tube	2129 reflections with *I* > 2σ(*I*)
graphite	*R*_int_ = 0.030
ω scans	θ_max_ = 27.5°, θ_min_ = 2.1°
Absorption correction: multi-scan (SADABS; Bruker, 2001)	*h* = −9→9
*T*_min_ = 0.976, *T*_max_ = 0.977	*k* = −16→15
7804 measured reflections	*l* = −14→19

### Refinement

**Table:**

Refinement on *F*^2^	Primary atom site location: structure-invariant direct methods
Least-squares matrix: full	Secondary atom site location: difference Fourier map
*R*[*F*^2^ > 2σ(*F*^2^)] = 0.046	Hydrogen site location: inferred from neighbouring sites
*wR*(*F*^2^) = 0.112	H atoms treated by a mixture of independent and constrained refinement
*S* = 1.03	*w* = 1/[σ^2^(*F*_o_^2^) + (0.0459*P*)^2^ + 0.2867*P*] where *P* = (*F*_o_^2^ + 2*F*_c_^2^)/3
3041 reflections	(Δ/σ)_max_ < 0.001
201 parameters	Δρ_max_ = 0.18 e Å^−^^3^
4 restraints	Δρ_min_ = −0.25 e Å^−^^3^

### Special details

**Table:**

Geometry. All e.s.d.'s (except the e.s.d. in the dihedral angle between two l.s. planes) are estimated using the full covariance matrix. The cell e.s.d.'s are taken into account individually in the estimation of e.s.d.'s in distances, angles and torsion angles; correlations between e.s.d.'s in cell parameters are only used when they are defined by crystal symmetry. An approximate (isotropic) treatment of cell e.s.d.'s is used for estimating e.s.d.'s involving l.s. planes.
Refinement. Refinement of *F*^2^ against ALL reflections. The weighted *R*-factor *wR* and goodness of fit *S* are based on *F*^2^, conventional *R*-factors *R* are based on *F*, with *F* set to zero for negative *F*^2^. The threshold expression of *F*^2^ > σ(*F*^2^) is used only for calculating *R*-factors(gt) *etc*. and is not relevant to the choice of reflections for refinement. *R*-factors based on *F*^2^ are statistically about twice as large as those based on *F*, and *R*- factors based on ALL data will be even larger.

### Fractional atomic coordinates and isotropic or equivalent isotropic displacement parameters (Å^2^)

**Table:**

	*x*	*y*	*z*	*U*_iso_*/*U*_eq_	
O1	0.1869 (2)	0.17985 (9)	1.00835 (8)	0.0519 (4)	
H1	0.2127	0.1524	0.9611	0.078*	
O2	0.3338 (2)	0.13791 (9)	0.75220 (8)	0.0490 (4)	
O3	0.07206 (17)	0.08252 (9)	1.30718 (7)	0.0407 (3)	
O4	0.3910 (2)	0.75061 (10)	0.34205 (9)	0.0532 (4)	
N1	0.29393 (19)	0.02521 (11)	0.90464 (9)	0.0348 (3)	
N2	0.3324 (2)	−0.02124 (11)	0.82441 (9)	0.0339 (3)	
N3	0.3613 (2)	−0.13153 (13)	0.50292 (10)	0.0496 (4)	
C1	0.2257 (2)	−0.00223 (12)	1.05635 (10)	0.0307 (4)	
C2	0.1816 (2)	0.10410 (13)	1.07253 (10)	0.0324 (4)	
C3	0.1288 (2)	0.13524 (13)	1.15565 (10)	0.0342 (4)	
H3	0.0991	0.2061	1.1657	0.041*	
C4	0.1205 (2)	0.06050 (13)	1.22317 (10)	0.0312 (4)	
C5	0.1644 (2)	−0.04517 (13)	1.20894 (11)	0.0370 (4)	
H5	0.1592	−0.0953	1.2549	0.044*	
C6	0.2154 (2)	−0.07474 (13)	1.12659 (11)	0.0382 (4)	
H6	0.2442	−0.1458	1.1172	0.046*	
C7	0.2761 (2)	−0.03892 (14)	0.97002 (11)	0.0355 (4)	
H7	0.2962	−0.1114	0.9618	0.043*	
C8	0.3435 (2)	0.04041 (13)	0.75139 (10)	0.0335 (4)	
C9	0.3600 (2)	−0.01964 (12)	0.66533 (10)	0.0317 (4)	
C10	0.2673 (3)	0.01868 (15)	0.58540 (11)	0.0417 (4)	
H10	0.2037	0.0830	0.5847	0.050*	
C11	0.2709 (3)	−0.03991 (16)	0.50692 (12)	0.0501 (5)	
H11	0.2064	−0.0139	0.4538	0.060*	
C12	0.4556 (3)	−0.16551 (15)	0.57962 (12)	0.0442 (5)	
H12	0.5238	−0.2280	0.5776	0.053*	
C13	0.4581 (2)	−0.11375 (13)	0.66161 (11)	0.0358 (4)	
H13	0.5244	−0.1414	0.7136	0.043*	
C14	0.0200 (3)	0.18921 (14)	1.32548 (12)	0.0460 (5)	
H14A	0.1199	0.2366	1.3168	0.069*	
H14B	−0.0087	0.1943	1.3871	0.069*	
H14C	−0.0864	0.2086	1.2849	0.069*	
H2	0.342 (3)	−0.0930 (8)	0.8225 (16)	0.080*	
H4A	0.380 (3)	0.7860 (16)	0.3910 (10)	0.080*	
H4B	0.476 (2)	0.7815 (17)	0.3165 (13)	0.080*	

### Atomic displacement parameters (Å^2^)

**Table:**

	*U*^11^	*U*^22^	*U*^33^	*U*^12^	*U*^13^	*U*^23^
O1	0.0907 (11)	0.0363 (7)	0.0306 (7)	−0.0014 (7)	0.0150 (7)	0.0072 (5)
O2	0.0739 (10)	0.0350 (7)	0.0413 (7)	0.0045 (6)	0.0206 (6)	0.0002 (5)
O3	0.0562 (8)	0.0416 (7)	0.0263 (6)	0.0017 (6)	0.0139 (5)	−0.0029 (5)
O4	0.0814 (11)	0.0405 (8)	0.0406 (8)	−0.0106 (7)	0.0193 (7)	−0.0080 (6)
N1	0.0376 (8)	0.0437 (8)	0.0241 (7)	−0.0035 (6)	0.0080 (6)	−0.0041 (6)
N2	0.0423 (8)	0.0373 (8)	0.0234 (7)	−0.0013 (6)	0.0093 (6)	−0.0038 (6)
N3	0.0564 (11)	0.0604 (11)	0.0340 (9)	−0.0128 (8)	0.0148 (7)	−0.0113 (7)
C1	0.0321 (9)	0.0351 (9)	0.0252 (8)	−0.0028 (7)	0.0045 (6)	−0.0011 (7)
C2	0.0401 (10)	0.0319 (8)	0.0248 (8)	−0.0056 (7)	0.0022 (7)	0.0034 (6)
C3	0.0454 (10)	0.0276 (8)	0.0297 (9)	−0.0012 (7)	0.0050 (7)	−0.0028 (7)
C4	0.0326 (9)	0.0382 (9)	0.0231 (8)	−0.0031 (7)	0.0048 (6)	−0.0021 (7)
C5	0.0497 (11)	0.0346 (9)	0.0279 (9)	0.0011 (8)	0.0095 (7)	0.0067 (7)
C6	0.0505 (11)	0.0308 (9)	0.0345 (9)	0.0045 (8)	0.0105 (8)	0.0007 (7)
C7	0.0401 (10)	0.0380 (9)	0.0289 (9)	−0.0008 (7)	0.0066 (7)	−0.0029 (7)
C8	0.0366 (9)	0.0356 (9)	0.0293 (9)	0.0004 (7)	0.0087 (7)	0.0002 (7)
C9	0.0349 (9)	0.0354 (9)	0.0262 (8)	−0.0042 (7)	0.0094 (6)	0.0010 (7)
C10	0.0488 (11)	0.0458 (10)	0.0313 (9)	0.0046 (8)	0.0081 (8)	0.0052 (8)
C11	0.0562 (13)	0.0653 (13)	0.0286 (10)	−0.0055 (10)	0.0042 (8)	0.0029 (9)
C12	0.0471 (11)	0.0433 (10)	0.0447 (11)	−0.0026 (8)	0.0166 (9)	−0.0090 (8)
C13	0.0364 (10)	0.0408 (9)	0.0312 (9)	−0.0027 (7)	0.0082 (7)	0.0002 (7)
C14	0.0585 (12)	0.0435 (10)	0.0387 (10)	−0.0069 (9)	0.0171 (8)	−0.0122 (8)

### Geometric parameters (Å, °)

**Table:**

O1—C2	1.3471 (19)	C3—H3	0.9300
O1—H1	0.8200	C4—C5	1.385 (2)
O2—C8	1.224 (2)	C5—C6	1.365 (2)
O3—C4	1.3583 (19)	C5—H5	0.9300
O3—C14	1.425 (2)	C6—H6	0.9300
O4—H4A	0.861 (9)	C7—H7	0.9300
O4—H4B	0.853 (9)	C8—C9	1.496 (2)
N1—C7	1.276 (2)	C9—C10	1.383 (2)
N1—N2	1.3791 (19)	C9—C13	1.384 (2)
N2—C8	1.339 (2)	C10—C11	1.377 (2)
N2—H2	0.903 (9)	C10—H10	0.9300
N3—C11	1.329 (3)	C11—H11	0.9300
N3—C12	1.332 (2)	C12—C13	1.375 (2)
C1—C6	1.390 (2)	C12—H12	0.9300
C1—C2	1.398 (2)	C13—H13	0.9300
C1—C7	1.444 (2)	C14—H14A	0.9600
C2—C3	1.386 (2)	C14—H14B	0.9600
C3—C4	1.376 (2)	C14—H14C	0.9600
			
C2—O1—H1	109.5	N1—C7—H7	119.1
C4—O3—C14	117.81 (13)	C1—C7—H7	119.1
H4A—O4—H4B	106.4 (17)	O2—C8—N2	124.03 (15)
C7—N1—N2	115.77 (14)	O2—C8—C9	121.38 (14)
C8—N2—N1	119.21 (14)	N2—C8—C9	114.52 (14)
C8—N2—H2	122.6 (15)	C10—C9—C13	117.94 (15)
N1—N2—H2	118.1 (15)	C10—C9—C8	118.47 (15)
C11—N3—C12	116.82 (15)	C13—C9—C8	123.55 (15)
C6—C1—C2	117.59 (14)	C11—C10—C9	118.96 (17)
C6—C1—C7	119.62 (15)	C11—C10—H10	120.5
C2—C1—C7	122.77 (14)	C9—C10—H10	120.5
O1—C2—C3	117.41 (15)	N3—C11—C10	123.61 (17)
O1—C2—C1	121.76 (14)	N3—C11—H11	118.2
C3—C2—C1	120.83 (14)	C10—C11—H11	118.2
C4—C3—C2	119.47 (15)	N3—C12—C13	123.90 (18)
C4—C3—H3	120.3	N3—C12—H12	118.1
C2—C3—H3	120.3	C13—C12—H12	118.1
O3—C4—C3	124.30 (15)	C12—C13—C9	118.71 (16)
O3—C4—C5	114.93 (14)	C12—C13—H13	120.6
C3—C4—C5	120.77 (15)	C9—C13—H13	120.6
C6—C5—C4	119.09 (15)	O3—C14—H14A	109.5
C6—C5—H5	120.5	O3—C14—H14B	109.5
C4—C5—H5	120.5	H14A—C14—H14B	109.5
C5—C6—C1	122.25 (16)	O3—C14—H14C	109.5
C5—C6—H6	118.9	H14A—C14—H14C	109.5
C1—C6—H6	118.9	H14B—C14—H14C	109.5
N1—C7—C1	121.88 (15)		

### Hydrogen-bond geometry (Å, °)

**Table:**

*D*—H···*A*	*D*—H	H···*A*	*D*···*A*	*D*—H···*A*
O1—H1···N1	0.82	1.92	2.644 (2)	146.
O4—H4B···O2^i^	0.85 (1)	2.07 (1)	2.924 (2)	176 (2)
O4—H4A···N3^ii^	0.86 (1)	1.97 (1)	2.832 (2)	178 (2)
N2—H2···O4^iii^	0.90 (1)	2.02 (1)	2.915 (2)	169 (2)

Symmetry codes: (i) −*x*+1, −*y*+1, −*z*+1; (ii) *x*, *y*+1, *z*; (iii) *x*, −*y*+1/2, *z*+1/2.
